# Immediate Root Canal Filling*This is an extract taken from a paper by Dr. Kells, which is published in full, with illustrations, in The Dental *Items of Interest*, for February, 1917.

**Published:** 1917-03

**Authors:** C. E. Kells


### IMMEDIATE ROOT CANAL FILLING.*

BY C. E. KELLS, D.D.S.

For thirty years or more the immediate filling of putrescent root canals with or without sinuses, as taught him by Cassius M. Richmond, has been the writer's practice, and the results have been quite satisfactory.

An upper first bicuspid in the mouth of a young girl showed from a skiagraph a considerable area of rarified bone at the apex of the root. The tooth was not giving any trouble, but a very large defective amalgam filling was removed, under which was found a dead pulp.

The root canals were immediately filled without any regard being paid to the conditions beyond the apex, and so far nothing has been heard from the tooth, although it has been under observation. She did *not go* to another dentist to have it extracted!

Eight years ago an upper central, lateral and cuspid were filled in this manner for a certain patient who said that the abscess upon the cuspid was of twenty-five years' standing. This was the only tooth of the three that had a sinus. It closed immediately after the root was filled. This winter the patient underwent a most thorough examination at the Johns Hopkins Hospital, and these teeth amongst others were skiagraphed there at the instance of the internist. The condition about the roots of these three teeth was reported to be absolutely satisfactory, and yet the treatment they received—immediate root filling—eight years ago, is now considered by Drs. Grieves and Rhein to be impossible. How can they reconcile their position with this clinical report? Have they themselves thoroughly

* This is an extract taken from a paper by Dr. Kells, which is published in full, with illustrations, in The Dental *Items of Interest*, for February, 1917.

tried out this method and failed, and if not, how can they judge it to be impossible?

These satisfactory clinical results, satisfactory to the operator, satisfactory to the patient, satisfactory to the internist of the Johns Hopkins Hospital, only corroborate the usual results obtained in our regular practice.

Many years ago the writer was in the office (in another city) of one of our very prominent root canal exponents, when he was giving an upper lateral an "endless chain" treatment.

"Why do you not fill that canal permanently now?" he was asked (he knew the questioner was an "immediate" operator), and the reply was, "I haven't got the nerve." And there you are! Had he had the nerve to fill the root canal then and there he very probably would not now be teaching his present method, but he still condemns methods never tried.

Nothing but chloropercha is mentioned as a root canal filling.

If any who now condemn the immediate filling of putrescent canals do so because they tried the process and failed, using guttapercha for the filling, possibly their failures are not to be wondered at.

The conditions attending root canals vary greatly, and the writer classifies these conditions, recognizes that no one material can be best adapted to all, and fills each class with the material apparently best suited to its individual condition. Does not that appear reasonable? And it just so happens that in his practice chloropercha is very seldom used, having proven in his hands the most undesirable of all materials for this purpose.

Root canals are, as a matter of fact, treated just as are ordinary crown cavities. In each case the cavity or root canal, whichever it happens to be, is *cleaned* out, *sterilized* (?), *dried* and filled with the material selected as best for the case. In rare instances, very rare, a cavity can not be kept dry, and it is filled with what used to be called a "submarine filling." In rare instances, also very rare, a root canal *can not* be dried, and it likewise receives a submarine filling. Times are when we do not succeed in curing an abscess. This would appear to be a confessional, but it is not. It is merely a frank recital of facts, frankness being necessary in a discussion of this character, and no claim is made for one hundred per cent. of successes.

No one who has not tried immediate root canal filling and failed *conspicuously* has a right to condemn the method, and then he can only condemn it as practiced by himself. Roots so treated that have stood the acid test—that of durability—are indisputable evidence that the practice is sound, and even if, after only eight years, the results thus obtained are passed by the Johns Hopkins Hospital, what more need be said?

The writer's technic of root canal work, of course, is not touched upon here, and for two reasons:

1. It would take up too much space; and

2. From the tone of apparently *all* other writings upon the subject, they are entirely out of line, and would not interest the readers of the *Dental Items of Interest.*

Aseptic root canal procedure, *as it is called,* now holds the center of the stage. This does not appeal to the writer, because he believes that such work as performed is by no means aseptic, and that no work can or *should* be done in a truly aseptic manner upon a putrescent pulp. Let anyone who thinks otherwise first carefully note the treatment an *abscessed tooth* receives from the first to last by any aseptic (?) operator, and then go into any modern surgical amphitheatre and see how a real aseptic surgeon handles a case, and he will at once be convinced that the treatment of putrescent pulps in an aseptic manner is an impossibility. We must appreciate that a putrescent pulp in a tooth is an entirely different proposition from anything any general surgeon meets, and consequently the methods he uses *may not* be applicable to it, and, as a matter of fact, they are not.

It has been suggested that to advocate antiseptic root canal work "would be to plant ideas in the minds of men that slovenly work in the canal will do, because an antiseptic will follow up and take care of everything."

To take such a position is simply absurd. There is no such thing as slovenly work, *per se*. It is the operator who is slovenly. No slovenly operator can be transformed into what is considered an aseptic root canal operator by becoming obsessed with the aseptic fad, nor on the other hand will any good and conscientious operator slight his root canal work because he believes antisepsis is more practical.

Undoubtedly the majority of all dental operations performed today are not really well done.

Whenever an antiseptic root canal operator has failed it has been due either to the inefficiency of the operator or the attempt to accomplish the impossible, for not all "dead" teeth can be saved.

If, therefore, the inefficient root canal worker turns to asepsis to help him out of his troubles he will undoubtedly find there is "nothing in it" for him; while on the other hand the efficient antiseptic operator will probably meet with no handicap should he follow the trend and adopt the method now so erroneously called "aseptic root canal procedure."

The writer still adheres to antiseptic methods upon putrescent pulps, because they have proven satisfactory in the past; because real aseptic work upon putrescent pulps is out of the question with him; because he does not believe a cross between the two advisable, except from a spectacular point of view, and because he does not believe in spectacular stunts (that is, hardly ever), and which, by the way, do not affect root canal filling one way or the other.

